# Researches and Observations on the Causes of Scrofulous Diseases

**Published:** 1845-01

**Authors:** 


Art. I.—*Researches and Observations on the Causes of Scrofulous Diseases.* By J. G. Lugol, Physician to the Hospital St. Louis, &c. *Translated from the French, with an Introduction, and an Essay on the Treatment of the principal varieties of Scrofula.* By W. H. Ranking, M.D. Cantab, Physician to the Suffolk General Hospital.—*London*, 1844. 8vo, pp. 306.

We gave so full an analysis of the original work of M. Lugol, in our last Number, that any reference to its contents or general character, would be here quite out of place. We feel it, however, due to the translator to say a few words respecting the treatise in its present form.

Although the impression conveyed by our former judgment of M. Lugol's book, must have been, on the whole, unfavorable, we would here explain, that our verdict was as much influenced by what the author had failed to do as by what he had done in regard to matters of fact and science; and as much by his unphilosophical mode of conducting the inquiry, as by the actual matter contained in the volume itself. We did not and do not think the book absolutely but only relatively bad. We thought and think that a much better book might have been written by one possessed of the author's great experience, or by the author himself if he had been better disciplined in inductive philosophy. But still we thought and think that,—whether viewed abstractedly as a mere book medical, or relatively as one among the hundreds annually published,—it must be regarded as far above par in actual value. It was, therefore, with much satisfaction that we learned that it was being translated; and it is with still greater satisfaction we are now enabled to announce that it has been admirably translated. The style bears none of the external marks of a translation, and reads as the original composition of a scholar and a man of taste.

The translator has prefixed an Introduction of some thirty pages, and has appended an essay on Treatment, extending to nearly a hundred. Both these are extremely well written, and will be perused both with pleasure and advantage by the English student. In conclusion, we heartily commend this volume to the notice of our readers. Whatever opinion they may have formed of the original work, they may depend on finding the present version, at least, better than its prototype,—thus, in itself, showing the reversal of a law which, we are sorry to say, too often governs the descent of books as well as of the malady whose history occupies its pages,—

"————nequiores, mox daturos
Progeniem vitiosiorem."

